# Effect of polyaniline content and protonating dopants on electroconductive composites

**DOI:** 10.1038/s41598-021-86950-4

**Published:** 2021-04-05

**Authors:** Katarzyna Bednarczyk, Wiktor Matysiak, Tomasz Tański, Henryk Janeczek, Ewa Schab-Balcerzak, Marcin Libera

**Affiliations:** 1 Institute of Chemistry, University of Silesia in Katowice, 9 Szkolna Str., 40-006 Katowice, Poland; 2 Institute of Engineering Materials and Biomaterials, Silesia University of Technology, 18A Konarskiego Str., 44-100 Gliwice, Poland; 3 Centre of Polymer and Carbon Materials, Polish Academy of Sciences, 34 M. Curie-Sklodowskej Str., 41-819 Zabrze, Poland

**Keywords:** Energy science and technology, Materials science

## Abstract

Elastic constructive elements prepared by electrospinning using polyacrylonitrile/polyaniline (PAN/PANI) electroconductive composites were prepared and investigated in terms of their thermal and mechanical properties. This study was focused on the impact of the type of counterion of polyaniline and the PANI content in composites on the thermal, conductive and morphological properties of electrospun fibers. In this study, composites obtained from PANI doped with sulfuric acid showed the highest conductivity, and composites obtained from PANI doped with hydrochloric acid showed the highest thermal stability. All obtained composites exhibited good thermal stability, with T_5_ values in the range of 230–268 °C that increased with increasing PANI content. The prepared composites exhibited comparable PAN T_g_ values, which indicates their suitability for processing. Instrumental analysis of polymers and composites was carried out using UV–visible spectroscopy, thermogravimetric analysis, differential scanning calorimetry, dynamic mechanical thermal analysis and scanning electron microscopy.

## Introduction

Electroconductive composites of polymers that can be processed by electrospinning to form elastic constructive materials are of major scientific and research interest for their application in innovative detectors, cells or diodes^1,2^. Conductive composites, due to their ability to be used as a building material, can be applied in electronic devices, such as batteries, capacitors, and photovoltaic cells. Assembly by electrospinning using conductive composites allows the production of elements for the construction of photonic devices, among others^3,4^.

Photovoltaics are one of the many areas of application for PANI nanofibers because this polymer is an attractive alternative to the platinum counter electrode for dye-sensitized solar cells (DSSCs) due to its lower cost of production. To date, many DSSCs in which the counter electrode was made of PANI or was supplemented with this polymer in nanoparticle form have been investigated, and the efficiencies of cells based on this type of material vary depending on the PANI form and admixture concentration (from 0.68 to 7.15%)^5–11^.

Polyaniline has been one of the most investigated conductive polymers in recent years due to its unsophisticated synthesis and good thermal stability^12^. Two major routes of PANI synthesis are chemical oxidative polymerization and electrochemical polymerization. The properties of polyaniline depend on the synthesis methodology and protonation state. The reduced form of polyaniline is leucoemeraldine, and the oxidized form is pernigraniline. The neutral form, which is half reduced and half oxidized, is emeraldine base. Proton-doped polyaniline, produced using acids, forms emeraldine salt, which is a highly conducting polymer^13^. The synthesis of emeraldine salt is most often accomplished by oxidative polymerization in acidic media (pH 1–3) using oxidants such as potassium dichromate and ammonium persulfate (APS)^14^.

The shape and size of polyaniline particles depend on the synthesis procedure. In the literature, a large variety of procedures are used to obtain a defined shape and size for polyaniline. PANI can be formed into particles, spheres, wires, tubes and fibers^15,16^. Among the different methods to generate PANI with specific morphologies, two main approaches can be distinguished based on the use of templates (hard-template, soft-template) or no templates in the syntheses^15–17^. The template-based method is associated with problematic removal of the template from the reaction and reduced conductivity of the obtained polymers^17^. Template-based synthesis usually provides PANI in the shape of rods and fibers, whereas template-free synthesis results in spheres. The shape of the polymerization product depends strongly on the pH of the solution. Nanotubes are produced in weakly acidic media^18,19^, while nanofibers are produced in alkaline media^20^. In oxidative polymerization, the shape depends on the molar ratio of acid to aniline (Ani). In the case of hydrochloric acid (HCl) applied in a 1:1 ratio to Ani, the products are most often spheres and fibers with an average length of 1.2 µm. A change in the molar ratio of HCl to Ani to 3:2 results in a polymer shaped as microsized flakes without any regular morphology. Meanwhile, the product of synthesis conducted with a 2:1 molar ratio of HCl to Ani were flakes and fibers with an average length of 250–300 µm. Increasing the molar ratio to 4:1 produced fibers; these fibers can be 5 cm long when the molar ratio during synthesis is 8:1. The fiber diameter differs with the molar ratio of ammonium persulfate to Ani. With an increasing APS:Ani molar ratio, the size of the polymer decreases, and the shape changes from nanorods to nanoparticles^21^. The morphology of PANI particles also varies with the Ani concentration in the reaction mixture. Synthesis at a molar ratio of 1:1 and a lower (4.910 * 10^−2^ M) concentration of Ani shows a larger average diameter and better ordered fibers for the product than a higher concentration (7.810 * 10^−2^ M)^22^.

PANI has great application potential; however, due to its low solubility in ordinary organic solvents, its processing generates many problems. Accordingly, pure PANI nanofibers are rarely produced by electrospinning; instead, polyaniline is mixed with other polymers to produce a homogeneous spinning solution^23,24^. Such a procedure simplifies the electrospinning process and improves the mechanical properties of fibers^25,26^.

In 2009, Raeesi et al. presented a method of obtaining PAN/PANI nanofibers by electrospinning from a solution of PANI and PAN in N-methylpyrrolidone^5^. PANI that had been synthesized by oxidative polymerization in acidic medium was dissolved in NMP (N-metylo-2-pirolidon). A PAN mixture in NMP (20 wt%) was added to a solution of PANI in various amounts. The solutions for electrospinning varied in PANI concentration (10–30 wt%). The processing resulted in fibers with diameters ranging from 60 to 600 nm depending on the PANI concentration. The fiber diameter decreased with increasing PANI concentration. The conductivity of the prepared fibers, as expected, increased with increasing PANI concentration in the mixture.

In 2014, Zhang et al. described a method of electrospinning PAN/PANI nanofibers using a solution of polymers in N,N-dimethylformamide^6^. A morphology analysis of PANI/PAN composite membranes carried out by SEM confirmed that the fibers were highly porous and that changing the voltage during the electrospinning had a significant effect on the diameter of the fibers produced. As the voltage was increased, an increase in diameter from 125 to 222 nm was observed, which was the result of more charges being generated on the surface of the solution and better stretching of the polymer. In 2014, PANI/PAN nanofibers were produced by an electrospinning dimethyl sulfoxide:PAN:PANI solution^7^. The PAN concentration was kept constant at 7% by weight, while the PANI concentration was 1, 3 or 5% by weight relative to the weight of the PAN. Analysis of the morphology of the obtained conductive nanomaterials showed that as the concentration of conductive polymer increased, the fiber diameter, conductivity and elongation of the sample increased.

As an alternative to conventional electrode materials, stretchable conductive nanocomposites composed of conducting nanomaterials and elastomeric media have been extensively studied for stretchable interconnections and devices. These stretchable conductive nanocomposites consist of percolation networks of nanoscale conductive fillers in elastomeric matrixes.

DMTA is widely used for material analysis, especially to determine the viscoelastic properties of polymers. When the temperature is continuously changed, the material is exposed to an oscillatory force. The mechanical properties of polymers, such as the storage modulus (E′) and the loss modulus (E″), can be determined by dynamic mechanical thermal analysis (DMTA). The storage modulus represents the elastic properties, and the loss modulus represents the viscous properties of a material. The E″ to E′ ratio is the loss factor (tangent δ) and represents the damping properties of materials. The PANI emeraldine base shows relaxation in the range of − 90 to 65 °C, which is related to torsional motions of the aromatic ring in the chain^27^ or local motions of amine units^28^; a glass transition temperature of approximately 220 °C^29^; and a transition at approximately 180 °C that is related to crosslinking and decomposition of PANI. PAN has also been studied by DMTA, and in the regions of 80–100 °C and 140–160 °C, we can see transitions related to chain van der Waals mobility and nitrile group motions^30^.

The aim of the present work is to investigate the effect of counterions and the content of PANI on nanocomposites based on PAN.

## Experimental

### Materials

Aniline (99%, Sigma-Aldrich) was freshly distilled before polymerization under reduced pressure (7 mmHg). Ammonium persulfate (99%, Sigma-Aldrich), hydrochloric acid (99%, Sigma-Aldrich), acetic acid (99%, Sigma-Aldrich), sulfuric acid (99%, Sigma-Aldrich), methanol and potassium bromide (KBr, 99.9%, Avantor Performance Materials Poland S.A.) were used as received. Water was purified by distillation. Dimethylformamide (DMF, 99.8%) and polyacrylonitrile (Mw = 150,000 g/mol, 99%) were purchased from Sigma-Aldrich.

### Synthesis of polyanilines

Polyanilines were synthesized via oxidative polymerization in water with ammonium persulfate as the oxidant in a solution of hydrochloric acid (A), acetic acid (B) or sulfuric acid (C). The amounts of reagents in each synthesis are presented in Table 1. The reactions were conducted at room temperature (20 °C). Representative synthesis was accomplished as follows. Aniline (9.313 g; 0.1 mol) was poured into hydrochloric acid solution (100.0 g; 1.0 M) and stirred under ambient atmosphere (argon). A water solution (100.0 g) of ammonium persulfate (28.523 g; 0.125 mol) was added to the reaction mixture and then stirred for 3 h. The reaction mixture was filtered on filter paper, and dark-blue powder was collected after rinsing with a solution of acid as used for synthesis (0.1 M), water and finally methanol. The powder was dried at 60 °C in an oven under reduced pressure (12 h).

**Table 1:** Amounts of reagents used in the synthesis of polyaniline.

Polymer	Aniline (g)	Ammonium persulfate (g)	Acid	Reaction yield (%)
PANI A	9.313	28.523	Hydrochloric acid (A)	92
PANI B	7.672	23.480	Acetic acid (B)	87
PANI C	5.131	15.701	Sulfuric acid (C)	93

### Electrospinning

Representative solutions for electrospinning were prepared as follows: polyacrylonitrile (6.666 * 10^−2^ mmol) and polyaniline (0.1 g) were dissolved in dimethylformamide (10.0 g, 99.8%, Sigma-Aldrich). After 1 h of incubation, the solution was sonicated for 2 h prior to application to the pumping system of an electrospinning machine (FLOW—Nanotechnology Solutions Electrospinner 2.2.0–500). The final products were mixtures of DMF/PAN/PANI nanoparticles (sequentially (A), (B), (C)) at a concentration of 10% by weight and 1 and 3% concentrations of the PANI nanoparticles by weight. The process parameters are shown in Table 2.

**Table 2:** Parameters for electrospinning PAN/PANI solution.

Parameter	Value
**Electrospinning process parameters**	
Solution flow rate, p [mL/h]	3.5
Potential difference between the electrodes, U [kV]	20
Distance between the electrodes, d [cm]	12.5

### Characterization

Thermogravimetric analysis (TGA) measurements were performed using a Pyris 1 (Perkin Elmer, USA) device. Experiments were carried out in a nitrogen stream (20 mL/min) with a scanning rate of 10 °C/min in the temperature range of 30–900 °C. Differential scanning calorimetry (DSC) measurements were performed using a Q2000 calorimeter (TA Instruments, USA) in a nitrogen stream at a scanning rate of 20 °C/min. Samples were analyzed in aluminum pans in the temperature range of 0–180 °C. Fourier transform infrared (FTIR) measurements were performed in the range of 4000–400 cm^−1^ with potassium bromide (KBr)-pressed pellets using a Spectrum One instrument (Perkin Elmer, USA). The samples were measured at room temperature, and pellets were prepared by mixing 10.0 mg of polymer with 100.0 mg of KBr. The sample pellets were prepared by applying high pressure to a polymer sample with KBr. The spectral resolution was 4 cm^−1^ in transmittance mode. UV–Vis spectra of the PANI (4.0 mg) solutions in DMF (100.0 mL) were recorded using a Perkin–Elmer Lambda 40Bio spectrophotometer. The spectra were recorded from 250 to 900 nm. Scanning electron microscopy (SEM) measurements were performed using a scanning electron microscope (Quanta 250 FEG, FEI Company, USA) operating with an acceleration voltage of 10 kV under low vacuum (80 Pa). Electron micrographs were obtained from secondary electrons collected by a large-field detector (LFD). The samples were stuck to microscopic stubs by double-sided adhesive carbon tape. Micrograph analysis was carried out using ImageJ software (version: 1.52a, https://imagej.nih.gov/ij/download.html). Conductivity measurements were made using a two-point probe conductor using a Keithley 2400 multimeter. Sizes were determined with a Litesizer 500 (Anton Paar GmbH, Austria) equipped with a 658 nm laser. The measurements were carried out under at 90° in polystyrene cuvettes. Measurements were conducted 5 times at 25 °C for 60 s, with equilibration periods of 3 min. Dynamic mechanical measurements were carried out on a DMTA Q800 (TA Instruments, USA) analyzer. DMTA allowed us to investigate the mechanical property behaviors related to Brownian motion of the polymer chains, such as variation in the mechanical modulus versus temperature. The mechanical dissipation factor, tan δ, as a measure of the deformational energy dissipated as heat during each cycle, is given by tan δ = E″/E′. The storage modulus, loss modulus and tangent δ of the samples were determined at 1 Hz and from − 150 to 220 °C at a heating rate of 3 °C/min. The sample dimensions were 15.0 mm × 4.0 mm in compression mode. TGA, DSC, IR and UV–Vis as well as DMTA analysis were carried out using OriginPro 2019b (version: 9.6.5.169 x64, https://www.originlab.com/) software.

## Results and discussion

**Figure 1:**
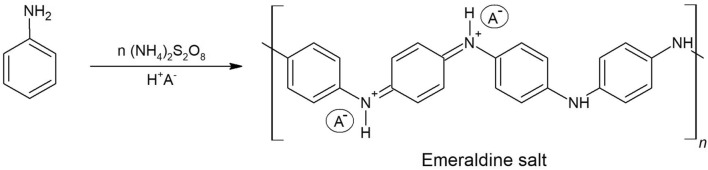
Oxidative polymerization of aniline using ammonium persulfate as an oxidant.

The syntheses of all polyanilines in acidic medium at room temperature yielded dark-blue powders. A scheme of oxidative polymerization is presented in Fig. 1. The reaction proceeded under oxidative conditions with ammonium persulfate, as usual for polyanilines^31^. The final counterions in the polyanilines were supplied using hydrochloric acid (PANI A), acetic acid (PANI B) and sulfuric acid (PANI C). After filtration, the powders were dried in a 60 °C oven under reduced pressure for 12 h and stored under an argon atmosphere. The reaction yields after filtration of powders were as follows: 92% for PANI A, 87% for PANI B and 93% for PANI C (Table 1).

The composite sheets that were the object of investigation were manufactured by applying electrospinning using the parameters listed in Table 2. Electrospinning conditions were selected on the basis of previous studies^5,32^. The materials were diversified by the counterion used for PANI synthesis and the PANI content in the composites. The composites were produced with electrospinning due to the superiority of this process over cast or bulk methods of preparation. As shown in the literature, electrospinning improves the flexibility and plasticity of thin films and membranes. Electrospinning also improves the mechanical properties of films^33^. Cast films show a higher degree of crystallinity and lower Young’s modulus values than electrospun films^34^.

**Figure 2:**
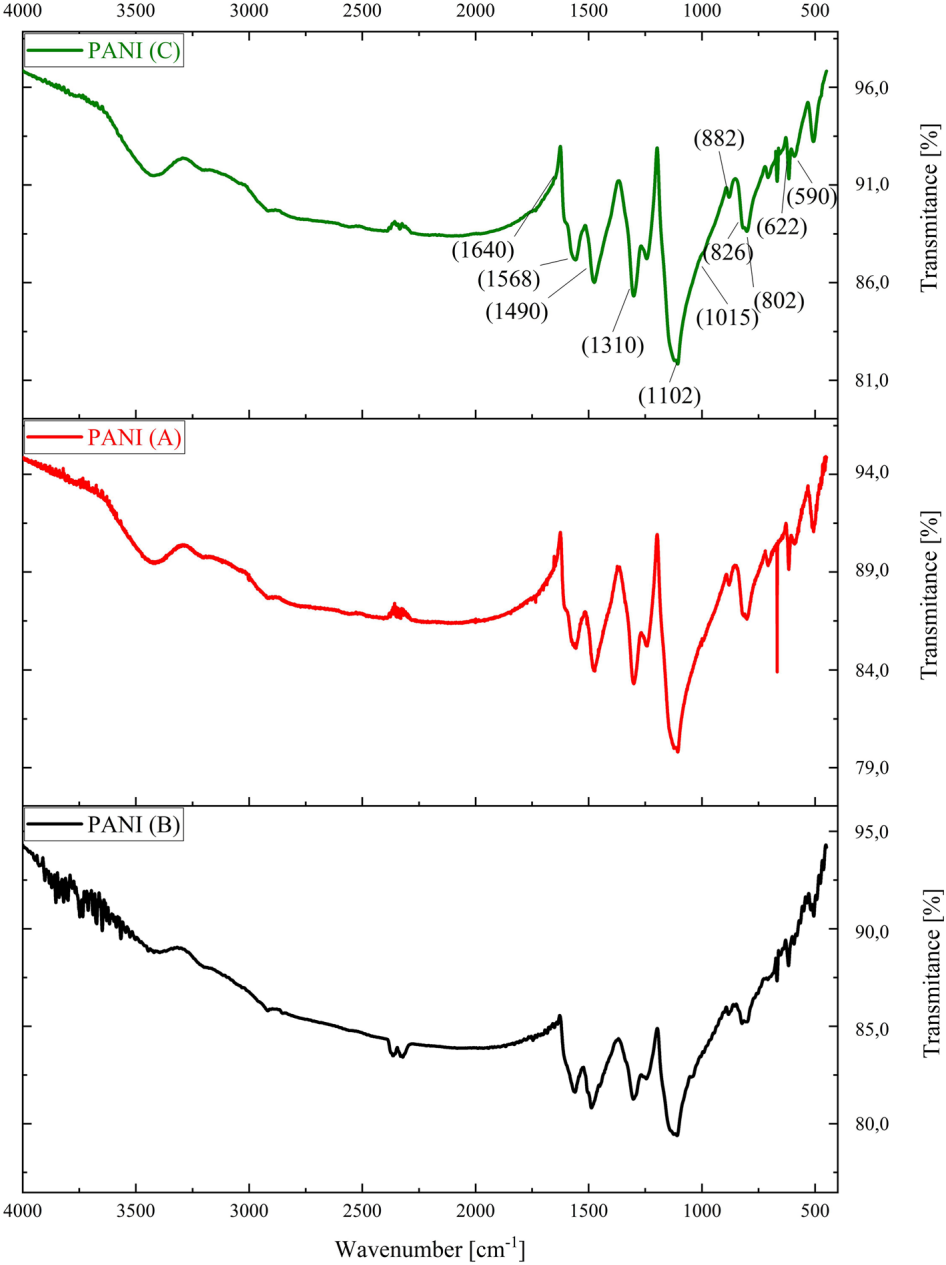
FTIR spectra of synthesized polyanilines (A, B, C).

The FTIR spectra (Fig. 2) of PANI A, PANI B, and PANI C reveal a N–H stretching band at 3400 cm^−1^. The quinonoid ring stretching vibrations are evidenced in the PANI A spectrum by the absorption band at 1568 cm^−1^, and the benzenoid ring stretching vibrations are at 1490 cm^−1^. The quinonoid ring stretching vibrations visible in the PANI B and PANI C spectra are represented by absorption bands at 1573 cm^−1^ and 1571 cm^−1^ and benzenoid ring stretching vibrations at 1497 cm^−1^ and 1494 cm^−1^, respectively. The shape of the 3400 cm^−1^ peak is the feature most dependent on the dopant because of the hydrogen bonding with amine groups. C–N stretching bonds are visible at 1102 cm^−1^, 1100 cm^−1^ and 1105 cm^−1^, and C=N stretching bonds occur in the spectra at 1640 cm^−1^, 1650 cm^−1^ and 1642 cm^−1^ for PANI A, PANI B and PANI C, respectively. The absorption band at 1310 cm^−1^ in the PANI A spectrum corresponds to π-electron delocalization induced in the polymer by C–H out-of-plane bending vibrations, which corresponds to a peak at 802 cm^−1^. In the PANI B spectrum, C–H out-of-plane bending was evidenced at 1307 cm^−1^ and 823 cm^−1^. The PANI C spectra exhibited C–H vibrations at 1305 cm^−1^ and 825 cm^−1^. The absorption at 1310–1305 cm^−1^ represents C–N vibrations of secondary aromatic amines. The peaks at 1015 cm^−1^ and 826 cm^−1^ are related to the substituted ring of polyaniline, the latter being in the substitution region (900 to 650) cm^−1^. The spectra show a band located at 1150 cm^−1^, which has been assigned to the vibration mode of the –NH^+^= structure and is associated with the vibrations of the charged polymer quinonoid structure. This indicates the existence of positive charges on the polymer chain. The shoulder observed at 1045 cm^−1^ for the PANI C spectrum is attributable to symmetric SO_3_ stretching in the hydrogen sulfate counterion. In spectra of PANI B and PANI A, there is no such peak. The bands at 882 cm^−1^ and 875 cm^−1^ in the PANI A and PANI C spectra, respectively, were attributed to acidic counterions. The bands observed at 590 and 629 cm^−1^ in the spectra also correspond to sulfate counterions. The bands observed at 580 and 640 cm^−1^ in the spectra also correspond to chloride counterions. The results confirm the structure of polyaniline presented in the literature^27–30,35–39^.

**Figure 3:**
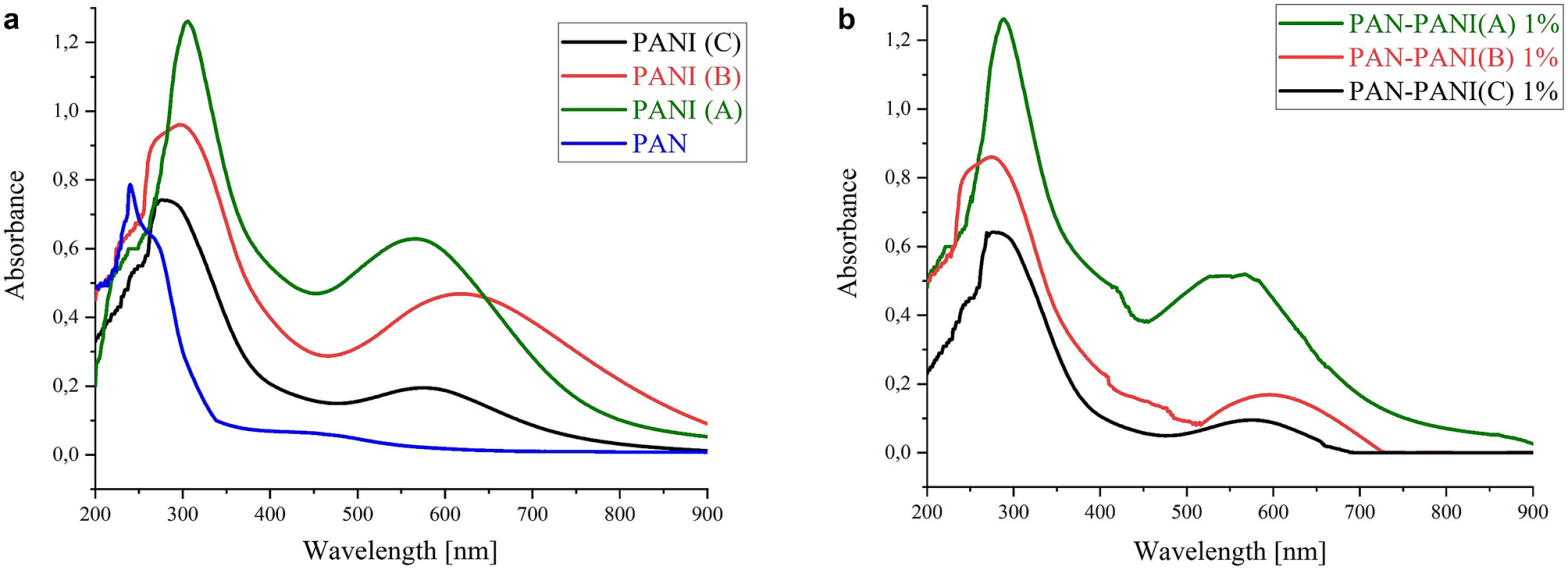
UV–Vis spectra of (**a**) synthesized polyanilines (A, B, C), polyacrylonitrile and (**b**) composites produced from polyanilines with polyacrylonitrile.

The UV–Vis absorption spectra of polyaniline (Fig. 3a) depend on the level of doping, extent of conjugation and solvent^40^. PANI spectra exhibit an absorption band at approximately 315–360 nm that is attributed to π–π* transitions in the benzenoid structure of the aromatic ring. The absorption band at approximately 580–630 nm corresponds to intramolecular transitions between the quinoid and benzenoid rings. The observed characteristic bands match those from the literature on PANI^41–43^. The UV–Vis spectra of the composites (Fig. 3b) exhibited absorption bands, and both bands shifted to the bathochromic region. The absorption bands at approximately 284–303 nm and 540–603 nm show lower intensities than those in the polyaniline spectra.

**Figure 4:**
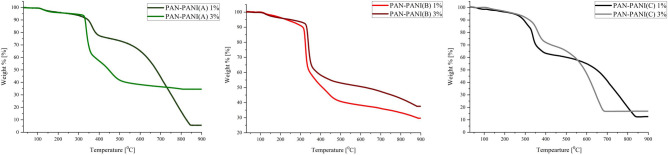
Thermogravimetric thermographs of the representative composites.

**Figure 5:**
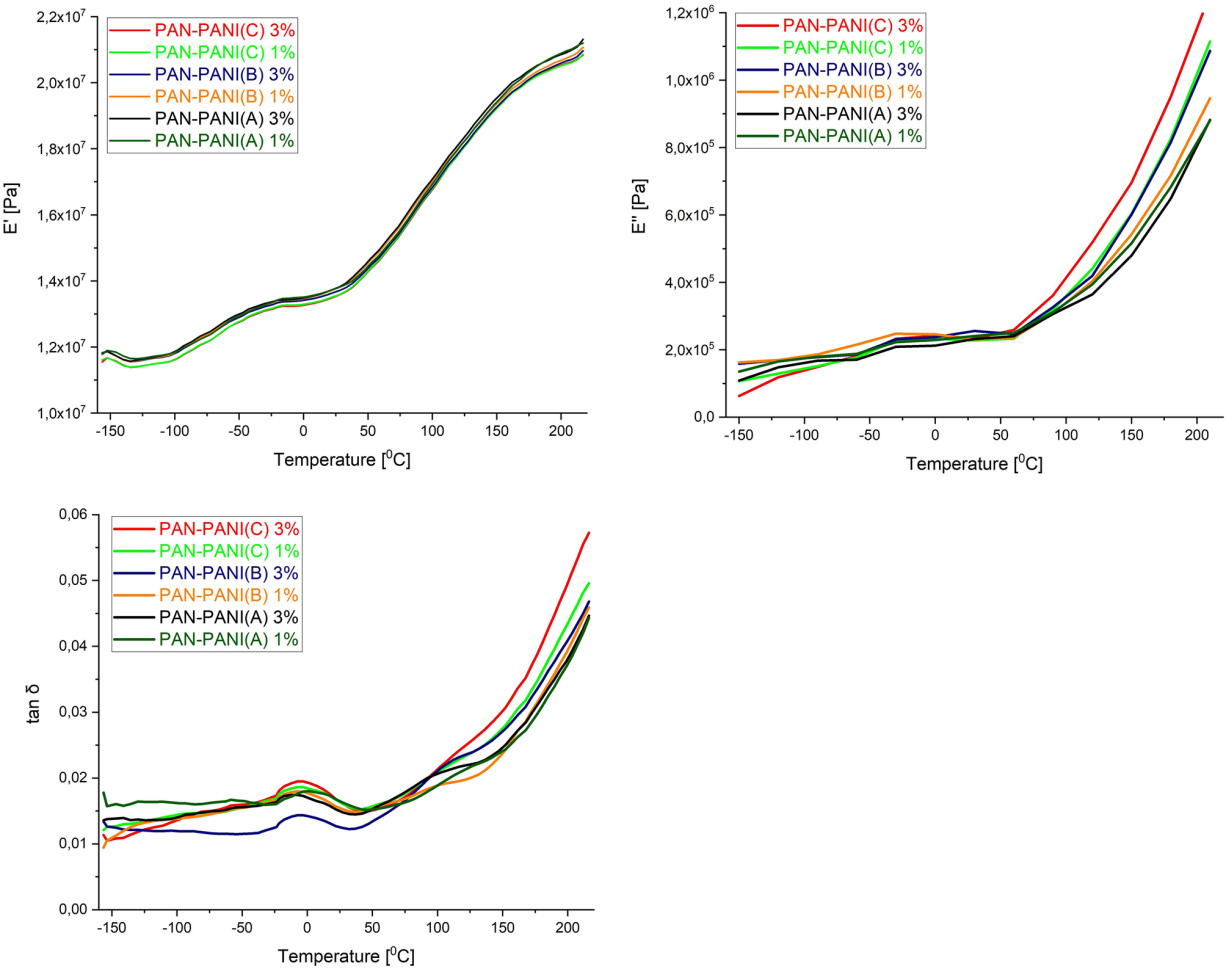
Loss modulus (E″), storage modulus (E′) and tangent delta (tan δ) of PAN–PANI composites.

The thermal properties of the synthesized PANI and the thermomechanical properties of the manufactured composites were analyzed using TGA, DSC and DMTA measurements. Thermal stability was characterized by the temperatures at which 5% (T5) or 10% (T10) weight loss occurred, which was defined as the temperature at the beginning of thermal decomposition, and by the temperatures at the maximum rate of compound degradation (Tmax), which were determined from differential thermogravimetric analysis (DTA). The glass transition temperature (*T*_*g*_) was characterized using DSC and DMTA measurements. The results obtained from thermal measurements are collected in Table 3, whereas those from thermomechanical measurements are collected in Supplementary Material. The TGA and DMTA curves are depicted in Figs. 4 and 5.

**Table 3:** Thermal properties of starting polymers and conductive composites.

Sample	T_5_ (°C)	T_10_ (°C)	Weight loss at 300 °C (%)	Residue at 900 °C (%)	T_max_ (°C)	T_gDSC_ (°C)	T_gDMTA_ (°C)
PANI A	71	149	17.7	0.4	69; 239; 312; 585	204	–
PANI B	45	136	32.4	5.4	43; 281; 579	205	–
PANI C	90	229	24.5	1.3	63; 222; 297; 586	206	–
PAN	321	325	0.8	37.1	325; 447	94	–
PAN–PANI A (1%)	258	346	6.4	8.6	44; 122; 336; 451	98	100
PAN–PANI A (3%)	268	331	5.6	34.6	32; 125; 368; 457	103	107
PAN–PANI B (1%)	230	311	8.9	29.6	38; 125; 326; 439	96	101
PAN–PANI B (3%)	251	330	6.5	37.5	44; 128; 338; 440	98	109
PAN–PANI C (1%)	244	293	11.5	12.6	70; 124; 170; 303; 337; 664	95	99
PAN–PANI C (3%)	252	320	7.9	19.9	50; 125; 305; 360; 772	98	106

T_5_ and T_10_ represent the temperatures of 5 and 10% weight loss, T_max_ is the temperature of the maximum decomposition rate as determined by DTA, T_gDSC_ is the glass transition temperature by DSC, and T_gDMTA_ is the glass transition temperature by DMTA.

The TGA thermograph of PAN shows a two-step degradation process. The first step of degradation indicates dehydrogenation of the polymer chain, and the second step indicates the depolymerization of the material (fragmentation of the macromolecule backbone), as reported previously^44^. Polyaniline shows three- or four-step degradation depending on the counterion. The first weight loss, varying in the range of 43–69 °C, is caused by the evaporation of residual solvent (methanol) used for precipitate purification after synthesis^25^. The second and third degradation steps occur in the range of 222–312 °C and are assigned to the degradation of polymer short fragments and the loss of dopant, respectively. PANI B exhibits a second step of degradation, which occurs between the second and third degradation steps for PANI A and PANI C. This phenomenon was explained by taking the radius of the dopants into consideration. The selected counterions have the following radii: 184 pm for chloride (PANI A), 162 pm for acetate (PANI B) and 258 pm for sulfate (PANI C). PANI B was synthesized with the dopant of the smallest radius; therefore, such ions could form much stronger contact ion pairs with PANI than larger ions, such as those in PANI A and PANI C, whose equilibrium will be shifted toward separated ion pairs. Additionally, a smaller counterion could be better incorporated through the polymer backbone. The last degradation step, occurring above 579 °C, is triggered by thermal degradation of the PANI backbone and agrees with values presented elsewhere^43^. The maximum degradation step for the backbone occurs at the lowest temperature for PANI B. Considering the T_5_ and T_10_ temperatures, PANI doped with sulfuric acid exhibited the best thermal stability.

The shapes of the TGA thermographs for composites of PAN–PANI A, B and C were similar to the thermographs of conductive polymers (Fig. 4). TGA thermographs indicated that the composites had better thermal stability than PANI but worse thermal stability than the PAN used in their creation. The thermal stability of the prepared composites was determined by the content of PANI in the bulk of the composite. The TGA results also prove that the PANI content impacts the thermal stability of the composite. Increasing the PANI concentration in the composite increased the thermal stability of all composites. The first weight loss of the composites is attributed to the evaporation of solvent used for composite purification. The second weight loss (between 122 and 128 °C) of the composites is attributed to the degradation of oligomers. The third weight loss of PAN–PANI C(1%) is assigned to evaporation of the solvent (dimethylformamide) used for composite preparation. Next, the degradation temperature of the composites varied in the range of 303–368 °C, which was attributed to dehydrogenation of the polymer backbone and loss of the PANI dopant. PAN–PANI C showed two peaks in this region, which are attributed to the sulfate having the largest radius of the counterions and are more evident for the composite. The last weight loss temperature of the composites, which appeared above 439 °C, is attributed to degradation of the polymer backbone, and it was related to the polyacrylonitrile degradation temperature. The general thermograph shape was dependent on the amount of polyaniline in the composites. The first and second weight losses for the composites diversified by PANI content were slightly different (Fig. 4). However, the third and fourth weight losses were more affected by the PANI content in the composite. The third weight loss for 1% and 3% PAN–PANI (A) occurred at 336 °C and 368 °C, whereas for 1% PAN–PANI (B) and 3% PAN–PANI (B), it is visible at 326 °C and 338 °C. For 1% and 3% PAN–PANI (C), weight losses first occurred at 303 °C and 305 °C and then at 337 °C and 360 °C, respectively. The temperature shifts were 22 °C for PAN–PANI (A), 12 °C for PAN–PANI (B) and 2 °C and 23 °C for PAN–PANI (C). Such evidence indicates better thermal stability for composites with 3% PANI than for those with 1% PANI content. The composite manufactured with PANI C exhibited the largest difference between the 1% and 3% additions. The final residue after thermal analysis was in the range of 12–38% of the initial weight and was, in all cases, approximately 8% higher for composites with 3% PANI content than for other composites. The degradation temperature of polymers increased with increasing PANI content. Composite PAN–PANI (A) exhibited a degradation peak at 451 °C for 1% PANI content and 457 °C for 3% PANI content. The degradation for PAN–PANI (B) started at 439 °C and 440 °C for 1% and 3% PANI, respectively. The PAN–PANI (C) composite exhibited a degradation temperature of 664 °C for 1% PANI content and 772 °C for 3% PANI content. The composite prepared with PANI doped with sulfuric acid exhibited the most stable thermograph.

The glass transition temperatures of polyanilines shown in Table 2 range from 204 to 206 °C, which are typical T_g_ values for polyaniline^10^. The T_g_ for PAN is 94 °C, which is similar to values in the literature^45,46^. The glass transition temperatures for composites determined via the DSC and DMTA methods were in the range of 95–112 °C. DSC thermographs of all samples (PANI, PAN and composites) showed that during the first heating scan there was an endothermic peak in the range of 40–60 °C due to the elimination of moisture, which was also observed in the TGA thermographs. The second endothermic peak visible in the DSC thermographs of the composites appeared in the range of 95–103 °C and was related to the glass transition temperatures of the materials. The DMTA T_g_ values were slightly higher than those obtained with the DSC method. Differences between the two methods were common, as the exact position of Tg depends on the frequency used in DMTA, whereas in DSC, it depends on the heating rate used^45^. In this study, the DSC heating rate was 20 °C/min, whereas the DMTA heating rate was 3 °C/min. In general, the T_g_ occurs at higher temperatures for composites with 3% PANI than for composites with 1% PANI. For example, the PAN–PANI (A) 1% T_g_ obtained by DSC was 98 °C, while that obtained by DMTA was 100 °C. The PAN–PANI (A) 3% T_g_ obtained by DSC was 103 °C, while that obtained by DMTA was 107 °C. The difference between T_g_ for PAN–PANI (A) 1% obtained with the DSC method was 5 °C, and that obtained by the DMTA method was 7 °C. The PAN–PANI (B) 1% T_g_ values were 96 °C (DSC) and 101 °C (DMTA), and the PAN–PANI (B) 3% T_g_ values were 98 °C (DSC) and 109 °C (DMTA). The differences in T_g_ between PAN–PANI (B) composites with 1% and 3% PANI content were 2 °C for the DSC method and 8 °C for the DMTA method. The PAN–PANI (C) 1% T_g_ values were 95 °C (DSC) and 99 °C (DMTA), and the PAN–PANI (C) 3% T_g_ values were 98 °C (DSC) and 106 °C (DMTA). The differences in T_g_ values for this composite with 1% and 3% PANI content were 3 °C for the DSC method and 7 °C for the DMTA method.

The mechanical characterization of composites was performed in compression mode in the temperature range of − 150 to 220 °C at a heating rate of 3 °C/min and frequency of 1 Hz. The dimensions of the samples were 15.0 × 4.0 mm. Figure 5 shows the variations in the loss modulus, storage modulus and tangent δ of the materials. Table 4 shows the relationship of the storage modulus of the composites versus the temperature. Two peaks were clearly seen for the storage modulus: one in the − 100 to − 40 °C region (β) and another in the 20 to 70 °C region (β′). When the temperature rose above Tg, the storage modulus increased until 220 °C, which can be explained by physical and chemical crosslinking of the polyaniline chain^28,29,47–52^. The β transition between − 80 and − 60 °C is related to local motions of amine groups in polyaniline, and the β′ transition is related to benzene ring motions and rotations of amine groups, also as reported in the literature in the range of − 20 to 20 °C (β′). According to the literature, polyacrylonitrile shows its most intense transition at approximately 100 °C and a second minor transition at 150 °C. The lower transition temperature is characteristic of the paracrystalline phase transition of the polymer, and the higher corresponds to the amorphous phase transition (glass)^53^. Three curve inflections can be clearly seen in the loss modulus: the first is in the − 60 °C region, the second above − 30 °C, and the third above 60 °C. The peak starting to increase at 60 °C represents the glass transition of composites, in agreement with the tan δ results. The tangent δ curve indicates two glass transition temperatures, one at approximately − 10 °C and one that is related to DSC and DMTA measurements at 105 °C.

**Table 4:** Electric conductivity and fiber size of polymers and conductive composites.

Sample	Conductivity (S/cm)	Particle size in water (nm)	Yarn size (nm)
PANI A	8.7	49	–
PANI B	6.5	46	–
PANI C	10.8	57	–
PAN–PANI A (1%)	1.2 * 10^−2^	–	192
PAN–PANI A (3%)	1.2 * 10^−2^	–	221
PAN–PANI B (1%)	0.7 * 10^−2^	–	181
PAN–PANI B (3%)	0.9 * 10^−2^	–	211
PAN–PANI C (1%)	2.5 * 10^−2^	–	224
PAN–PANI C (3%)	2.8 * 10^−2^	–	234

β relaxation was observed for the PANI emeraldine base at − 80 °C (1 Hz)^28,48^. This relaxation is attributed to the torsional motions of the aromatic rings in the polymer chain, i.e., the torsions of benzenoid and quinoid cycles present in the structure of the chain. The β relaxation of polyaniline films in NMP (5 Hz) was also observed, in the region of − 80 to − 60°C^49^. These studies have shown that β relaxation is linked to the residual content of water in the films due to water molecules that are hydrogen bonded to the polymeric chain. Studies have noted β′ relaxation in polyaniline films in the region of − 20 to 2°C^49^. These studies explained the transition as being due to the motion of molecules hydrogen bonded to NMP.

The storage modulus value increases with increasing PANI in the composite, which can be attributed to the increased crosslinking of polyaniline with increasing temperature. The same trend is visible for the loss modulus and tan δ. The crosslinking restricts the mobility of the polymer molecules and, consequently, composite molecular mobility.

The results of conductivity measurements are given in Table 4. The measurements were repeated 10 times on each sample. The conductivity of the prepared composites was dependent on the PANI content and on the dopant used in the synthesis. The polymer prepared with acetic acid (PANI B) showed the lowest conductivity of all polyanilines, similar to the composite made from PANI B exhibiting the lowest conductivity of the composites. The highest conductivity was exhibited by the composite based on polyaniline doped with sulfuric acid (PANI C). In general, with more PANI, the composite material exhibits slightly better conductivity except for the material with PANI A. The literature suggests that such a conductivity value is promising for further investigations, that is, for this material’s use in electrode preparation for DSSCs^46^. The particle size of polyanilines was measured with dynamic light scattering in a water suspension at 25 °C. The polyaniline synthesized with sulfuric acid exhibited the largest particle size, whereas acetic acid doping revealed the smallest particle size for PANI.

**Figure 6:**
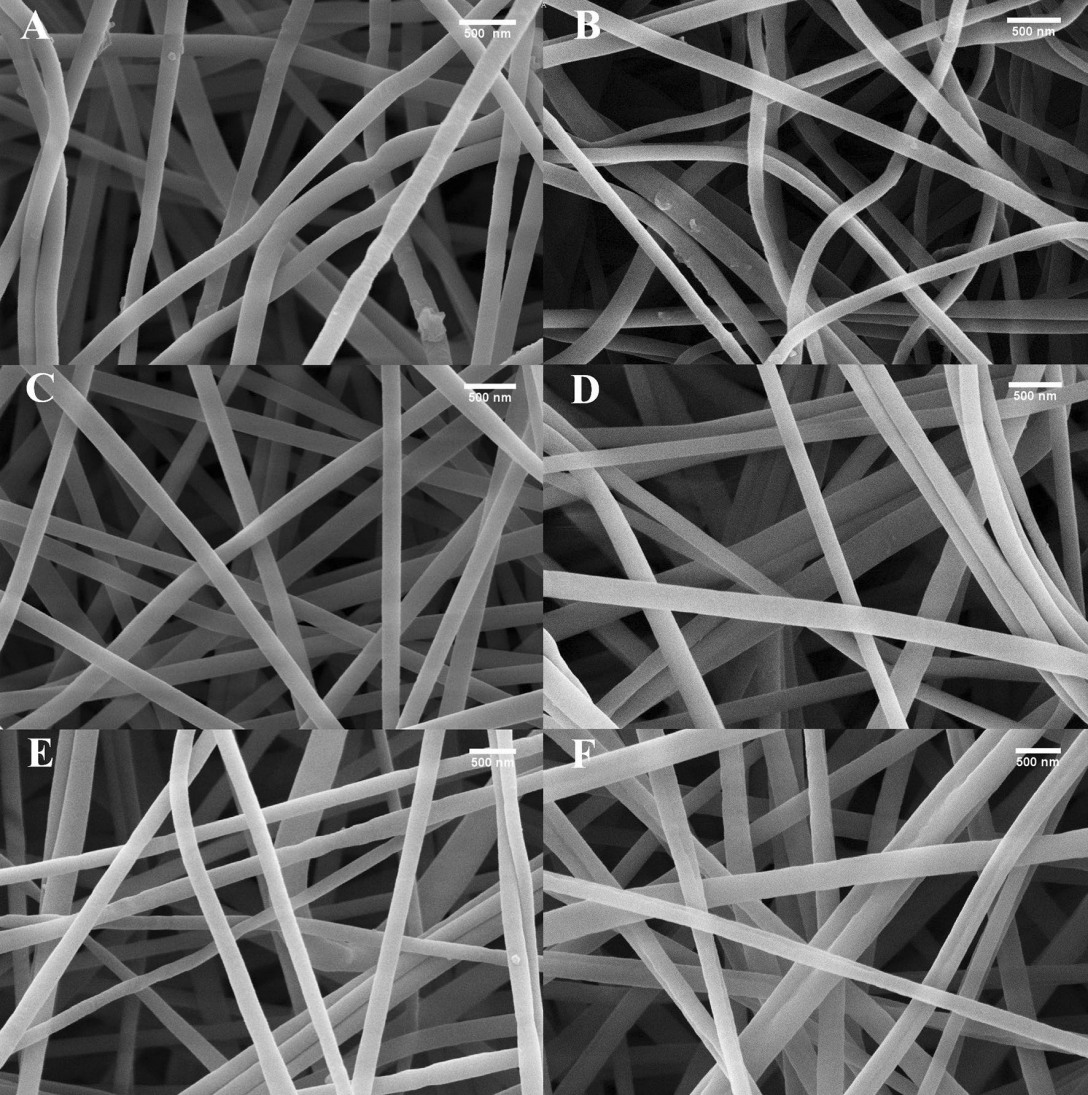
SEM photographs of polyaniline–polyacrylonitrile composites: (**A**) PAN–PANI A (1%), (**B**) PAN–PANI A (3%), (**C**) PAN–PANI B (1%), (**D**) PAN–PANI B (3%), (**E**) PAN–PANI C (1%), (**F**) PAN–PANI C (3%).

The SEM images presented in Fig. 6 show the surfaces of the polymer composites. The fiber diameter was averaged for 100 fibers from at least 5 micrographs. The results are shown in Table 4. As shown in Fig. 6, the composites have a three-dimensional network structure composed of randomly oriented polymer fibers. The yarn size is in the range of 192–234 nm and depends on the polyaniline content; thus, composites with 3% polyaniline content are slightly larger. For example, the PAN–PANI A (1%) composite exhibits a yarn size of 192 nm, and the same composite with a 3% PANI content exhibits a yarn size of 29 nm. In the case of the PAN–PANI B composite, the difference between yarn sizes of the 1% and 3% PANI contents was 30 nm, and that for the PAN–PANI C composite was 10 nm. The yarn size was related to the conductivity of the composites; the higher the conductivity of the material was, the greater the yarn size. Increasing the polyaniline content in the composite increased the formation of small agglomerates on fiber surfaces, which can be explained by the increasing conductivity of the composites. The yarn sizes were in agreement with the particle sizes determined by DLS in water. A dependence of particle size on yarn size was clearly visible. The largest particle size corresponded to the largest composite yarn. The PANI C polymer exhibited a particle size of 57 nm, and the composites showed sizes of 224 nm and 234 nm for the composite with 1% PANI and the composite with 3% PANI, respectively. PANI A was smallest PANI (49 nm), and PAN–PANI A (1%: 192 nm, 3%: 221 nm) was the smallest composite.

## Conclusions

Herein, the counterion impact on the thermal and mechanical properties of electrospun composite sheets was discussed. The explored materials were diversified by varying the counterion used for PANI synthesis and the PANI content in composite. The best thermal stability as represented by T_5_ was exhibited for 3% PANI obtained with hydrochloric acid; nonetheless, all samples were stable in the range of 230–268 °C. The thermal stability and glass transition temperature increased with increasing PANI content in the composite. The storage modulus similarly increased with increasing PANI content. Such variability can indeed be connected to the yarn size increasing with the content of PANI. The thermal stability of the composites predisposes them for use in the construction of organic electronics, among other applications. The impact of the PANI content on the electroconductivity of composites is less significant than the influence of the dopant type on the material properties. The highest conductivity (2.8 * 10^−2^ S/cm) was exhibited by composites based on polyaniline doped with sulfuric acid**.** It should be stressed that such composites seem to be promising for further study, i.e., for testing them as electrodes in dye-sensitized solar cells due to their costs of production being lower than that of platinum counter electrodes. Research involving modification of the electrospinning process and increasing PANI content will be developed in our further investigations.

## Supplementary Information

Supplementary Informations.
