# Supplementary material for: Effect of polyaniline content and protonating dopants on electroconductive composites
Source: Sci Rep. 2021 Apr 5;11:7487. doi: 10.1038/s41598-021-86950-4 (PMC8021568; doi:10.1038/s41598-021-86950-4)
Supplement: Supplementary file 1 — Supplementary Informations. [file 41598_2021_86950_MOESM1_ESM.docx]

**Effect of polyaniline content and protonating dopants on electroconductive composites**

Katarzyna Bednarczyk^a^, Wiktor Matysiak^b^, Tomasz Tański^b^, Henryk Janeczek^c^, Ewa Schab–Balcerzak^a,c^, Marcin Libera^a*^

*^a^Institute of Chemistry, University of Silesia in Katowice, 9 Szkolna Str., 40–006 Katowice, Poland,*

*^b^Institute of Engineering Materials and Biomaterials, Silesia University of Technology,
18A Konarskiego Str., 44–100 Gliwice, Poland*

*^c^Centre of Polymer and Carbon Materials, Polish Academy of Sciences, 34 M. Curie–Sklodowskej Str., 41–819 Zabrze, Poland*

**e–mail: marcin.libera@us.edu.pl, ORCID: 0000-0002-8786-2994**


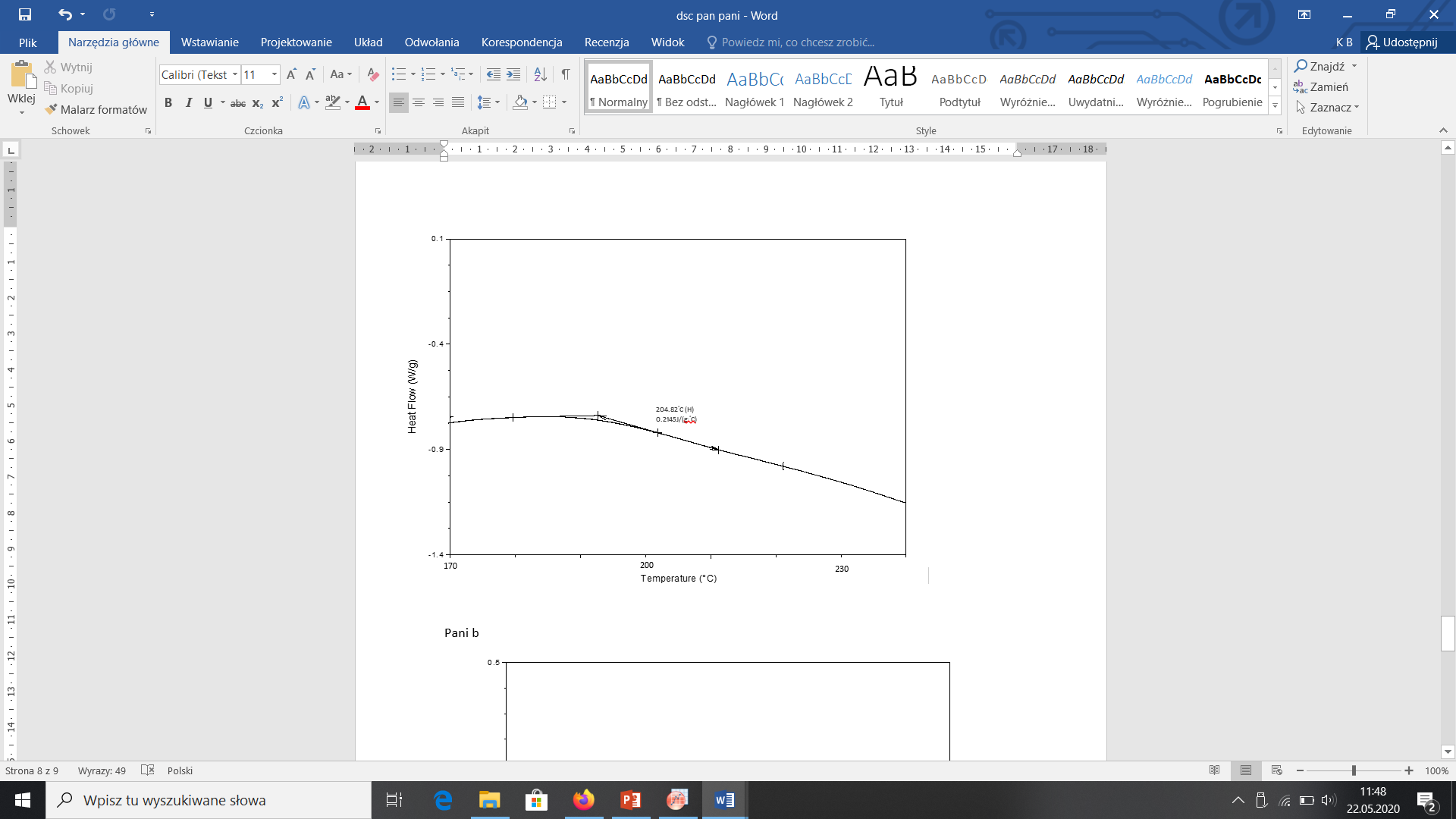

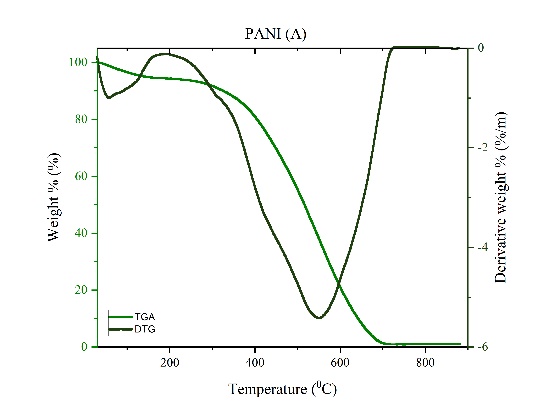


S 1 DSC and TGA graphs of PANI (A)


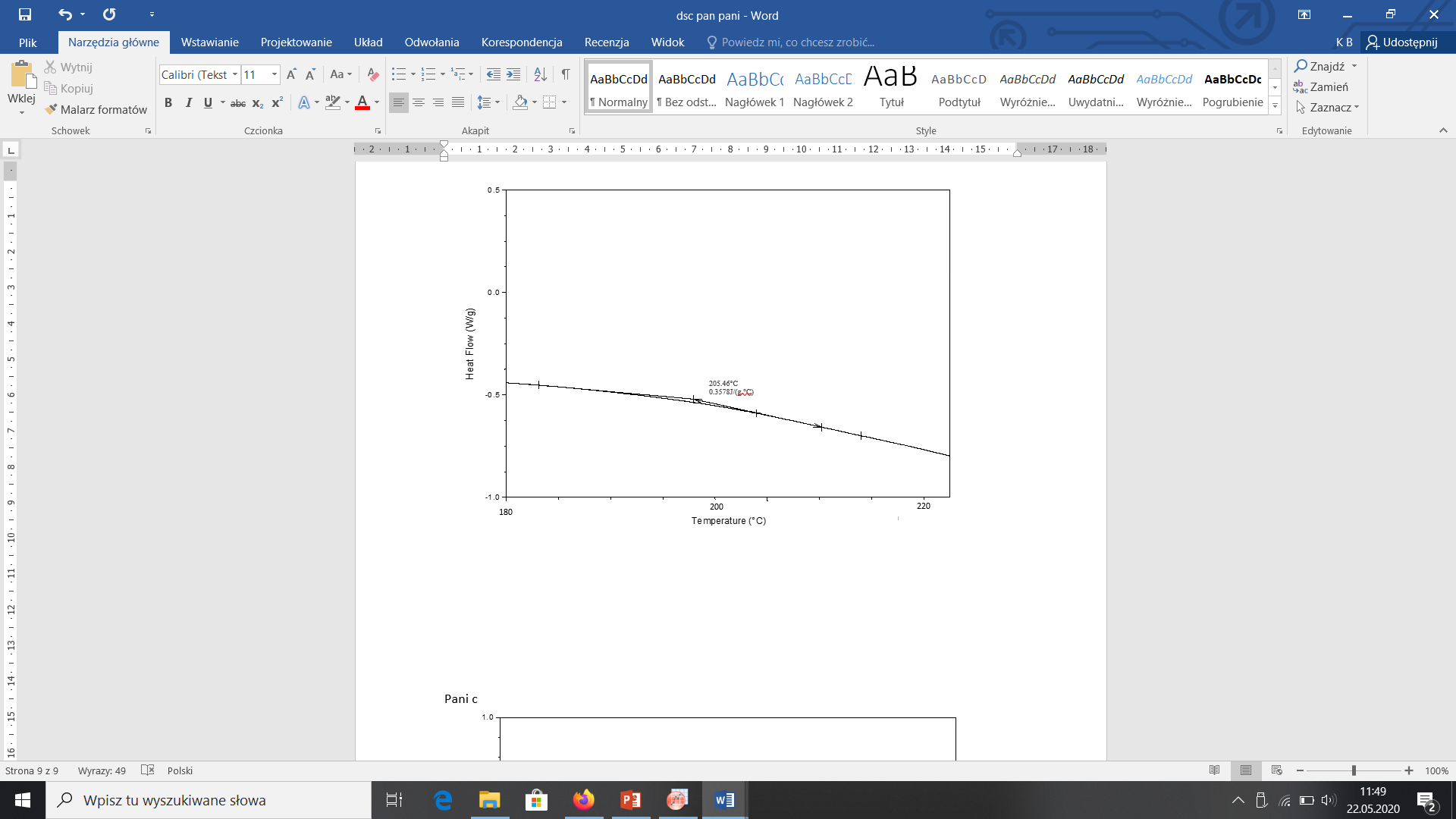

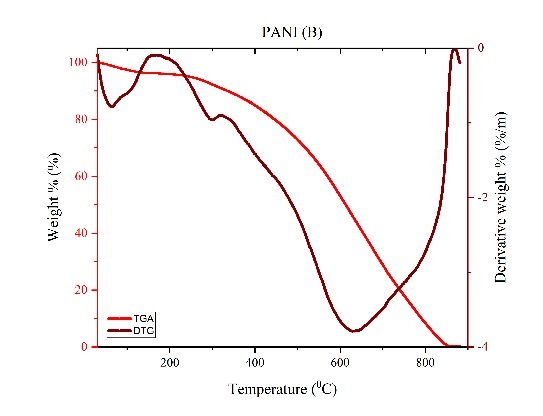


S 2 DSC and TGA graphs of PANI (B)


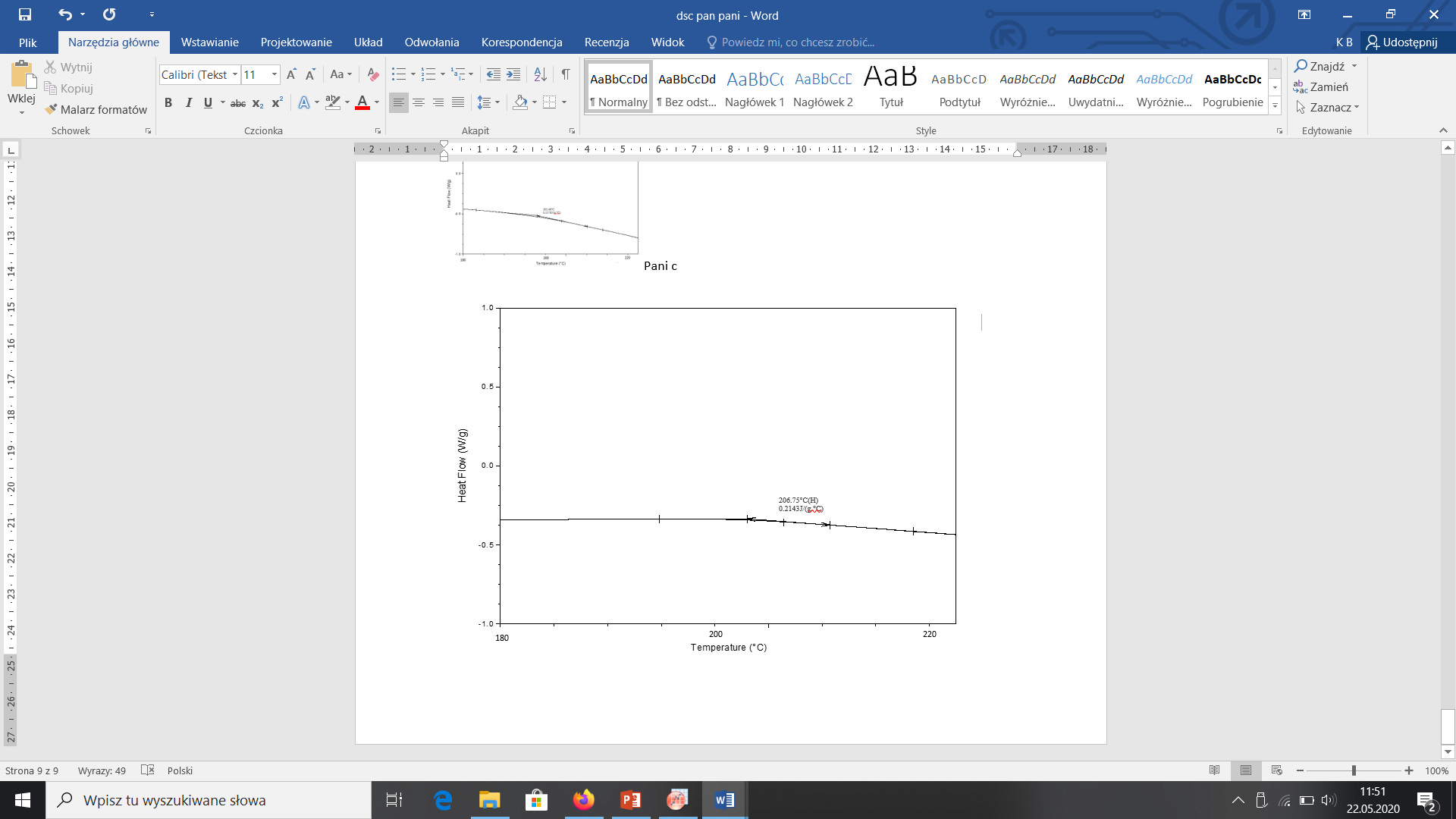

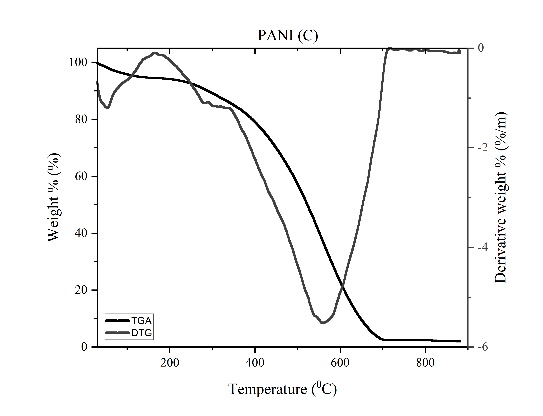


S 3 DSC and TGA graphs of PANI (C)


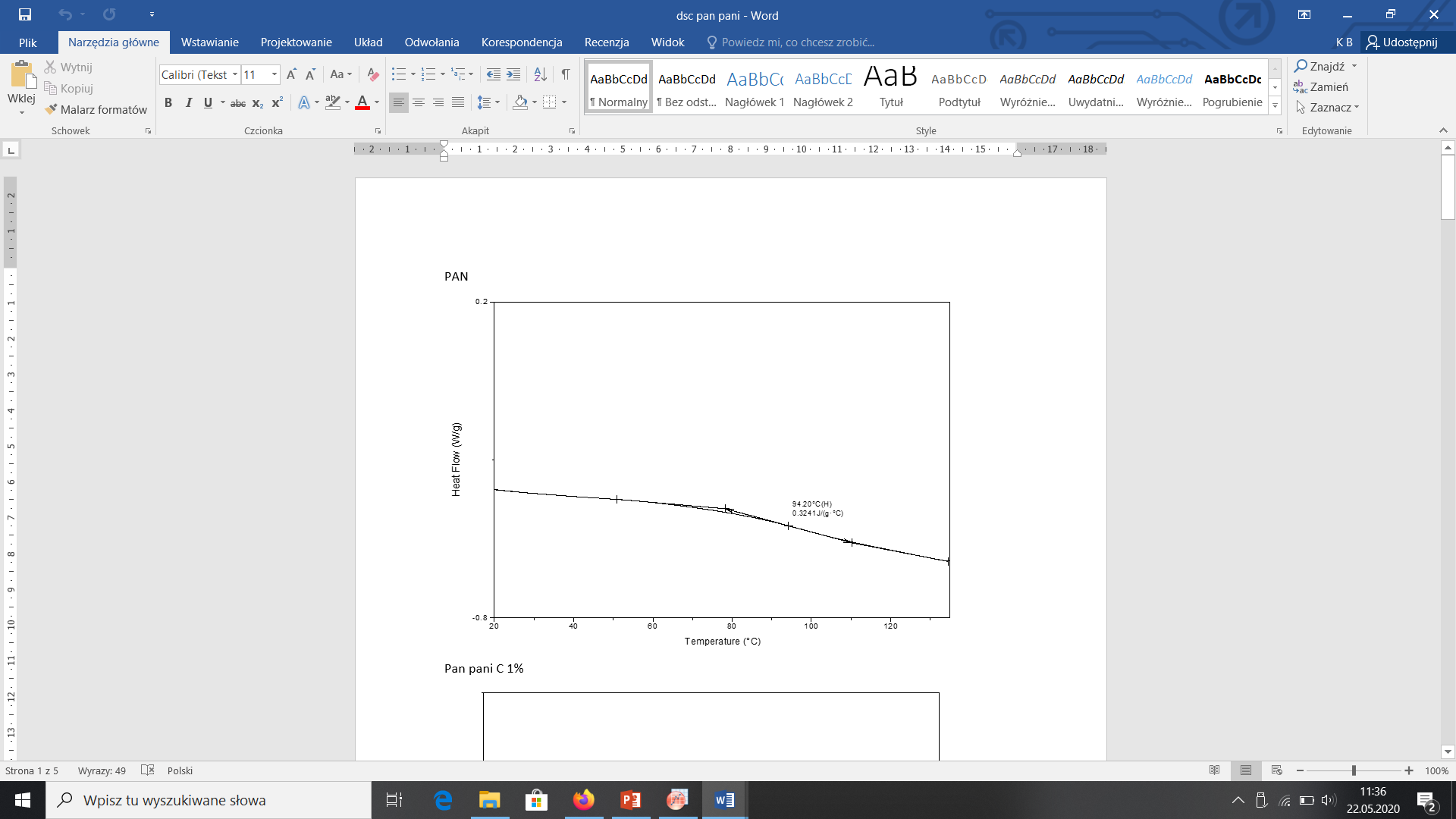

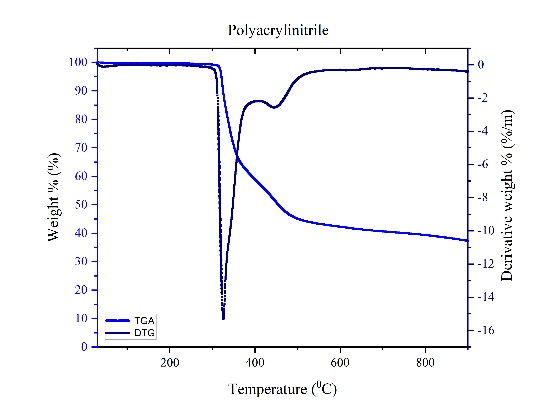


S 4 DSC and TGA graph of PAN


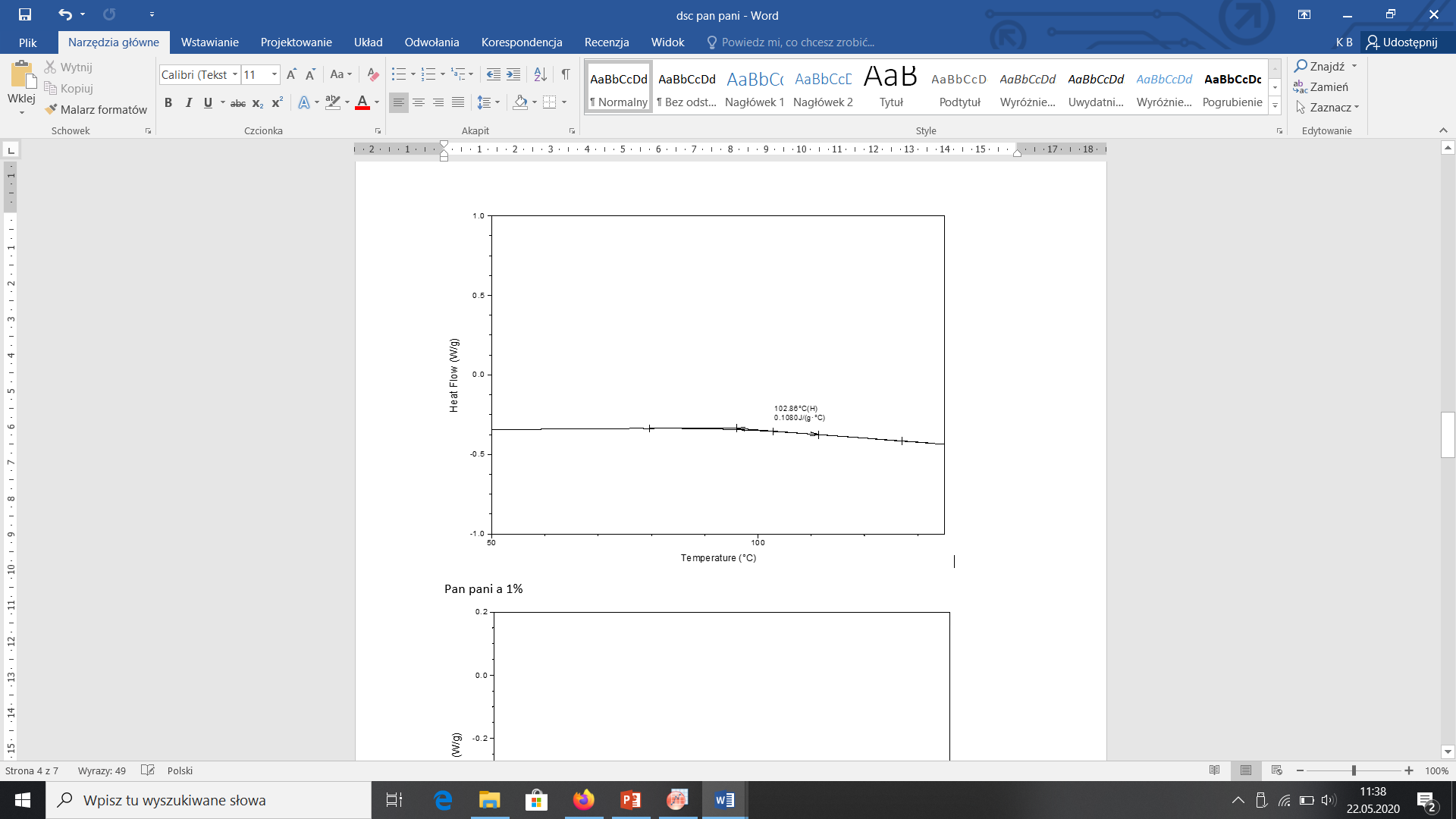


S 5 DSC graph of PAN-PANI (A)1%


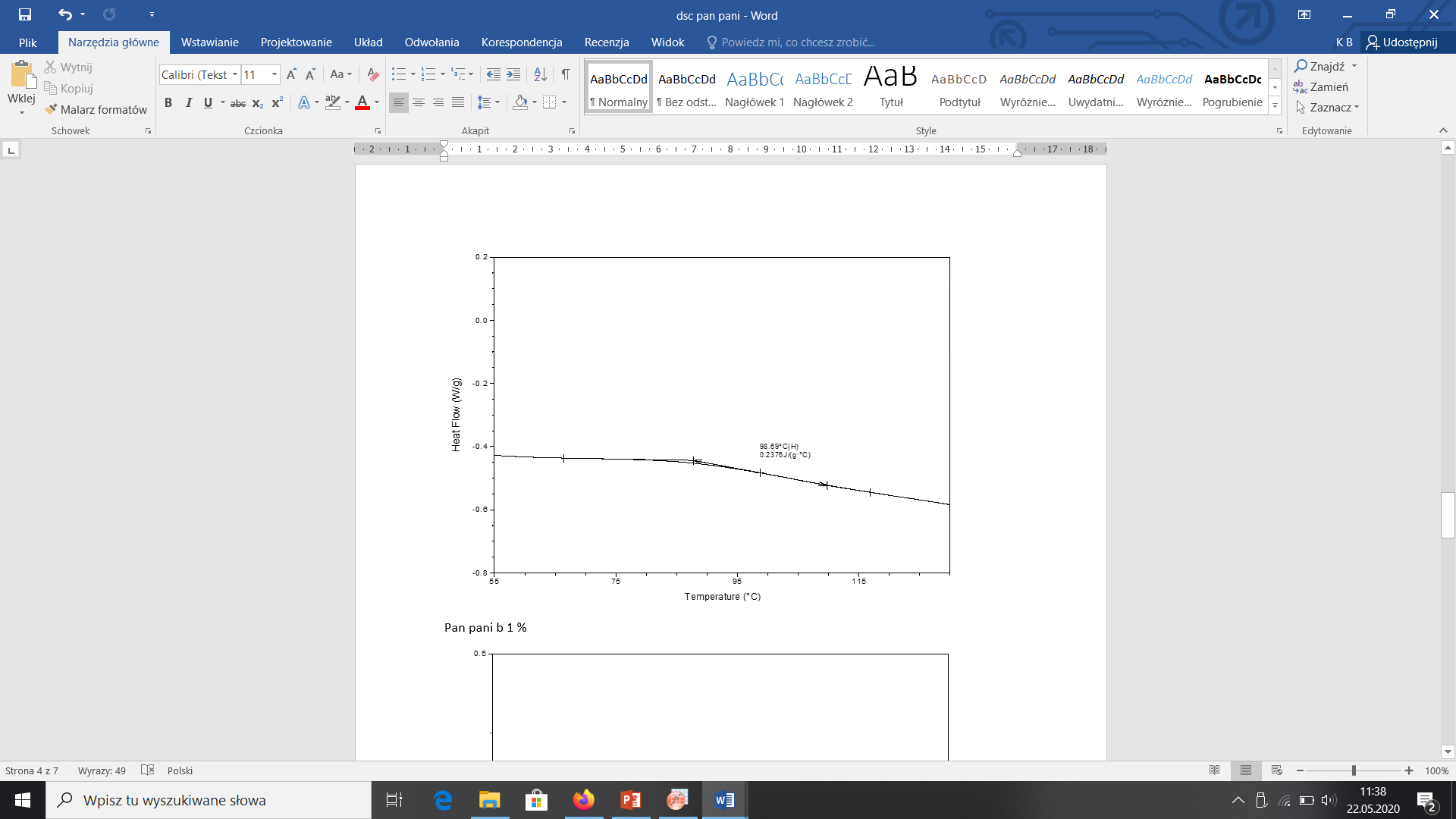


S 6 DSC graph of PAN-PANI (A)3%


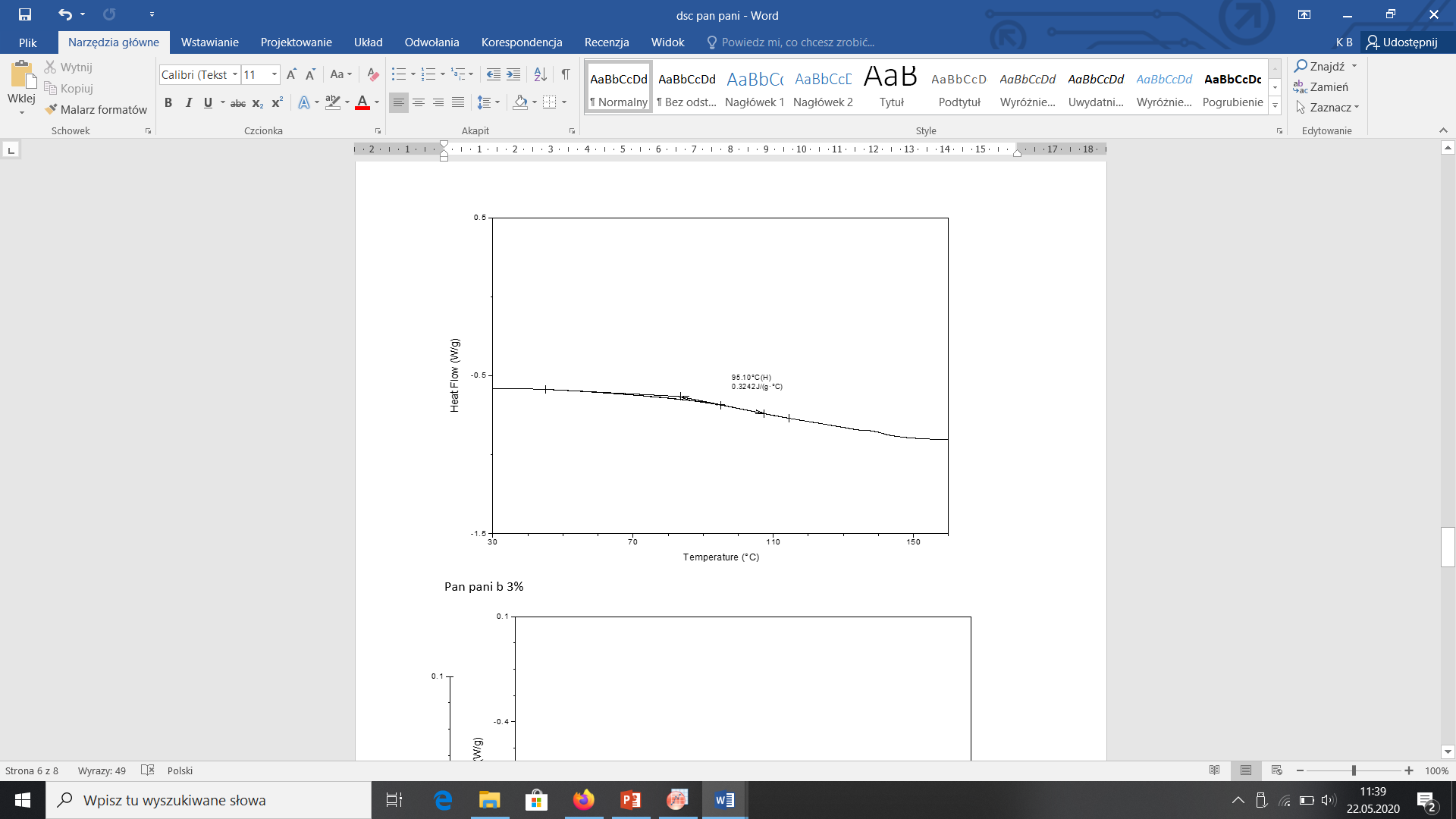


S 7 DSC graph of PAN-PANI (B)1%


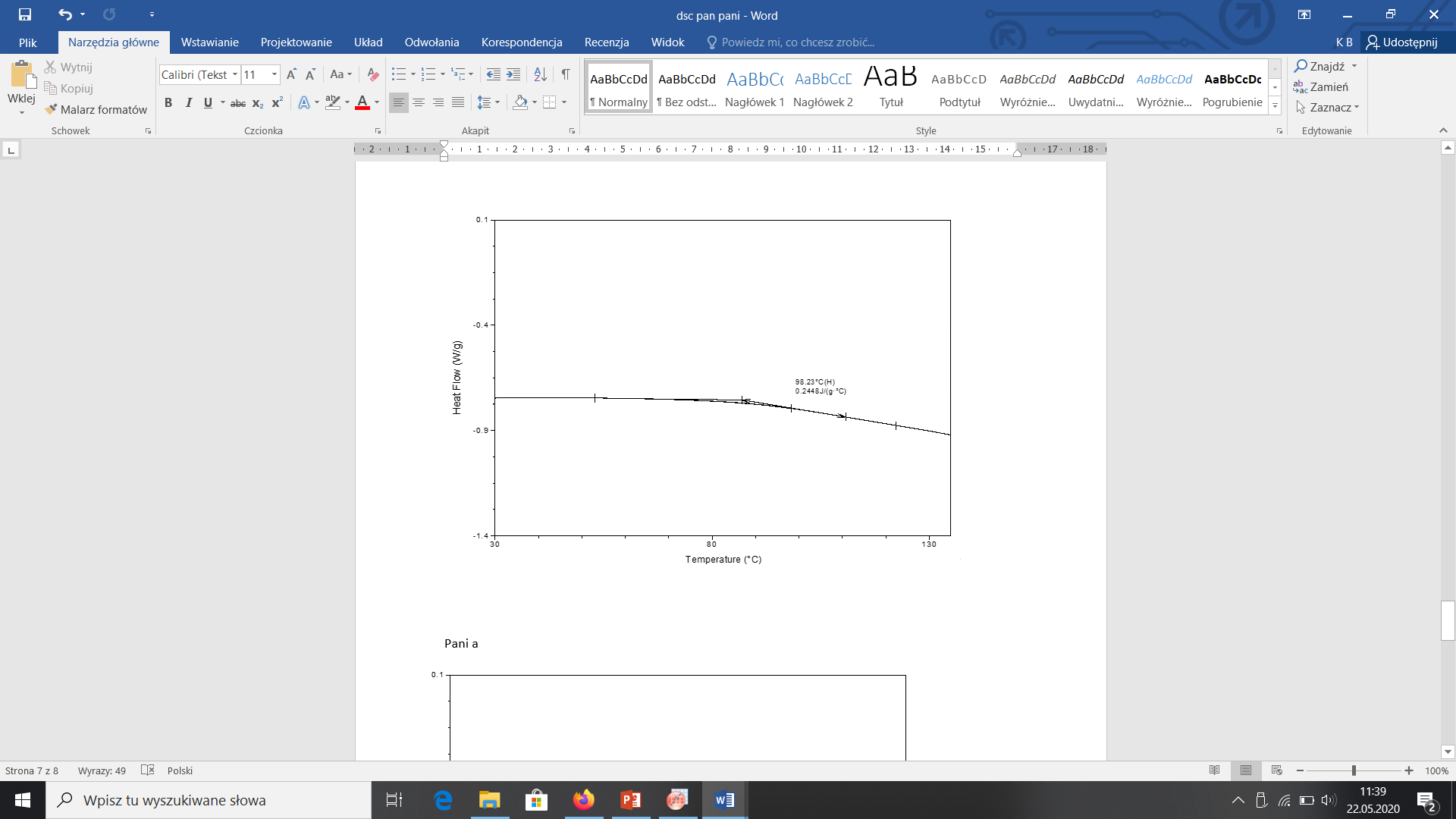


S 8 DSC graph of PAN-PANI (B)3%


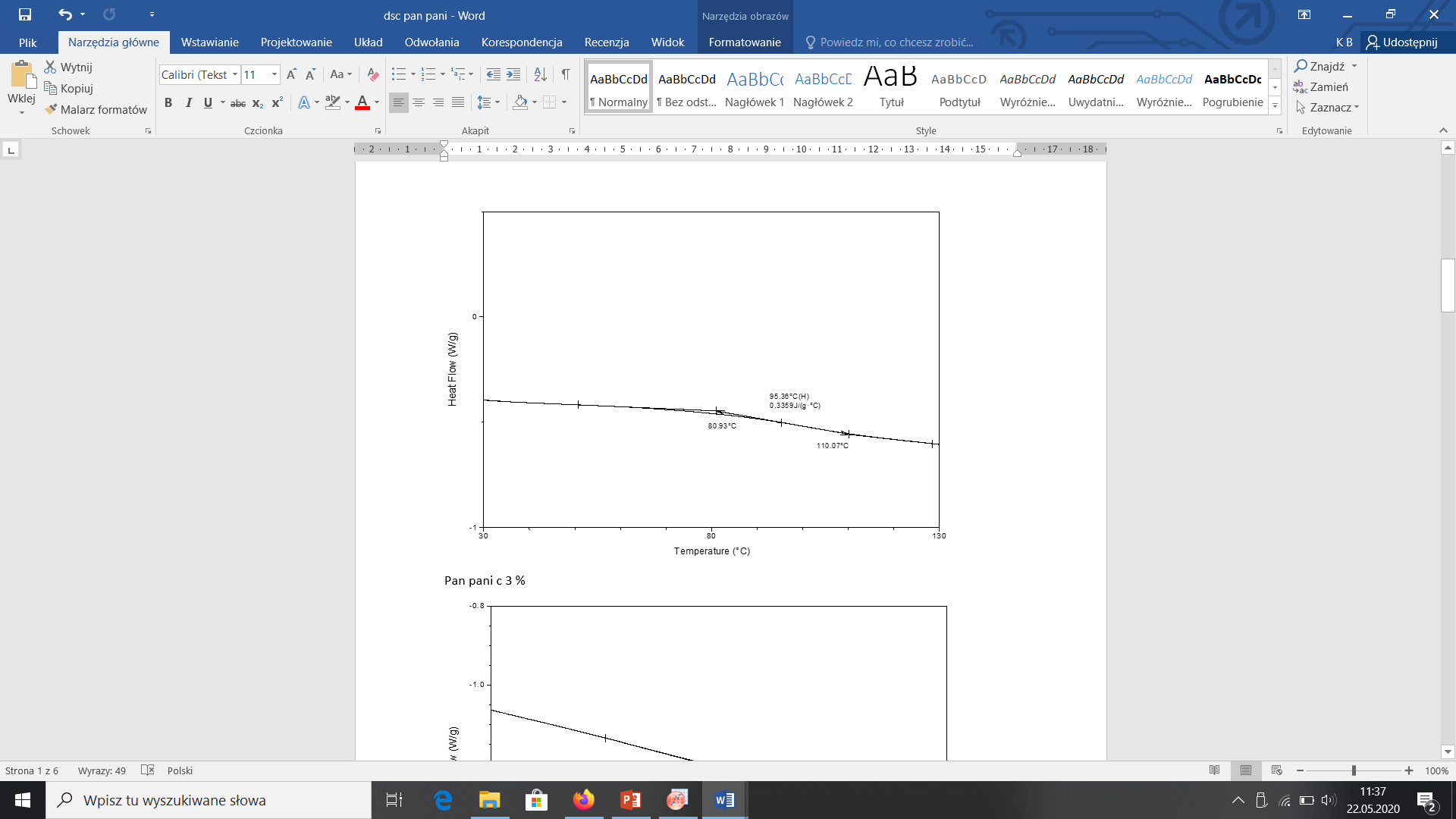


S 9 DSC graph of PAN-PANI (C)1%


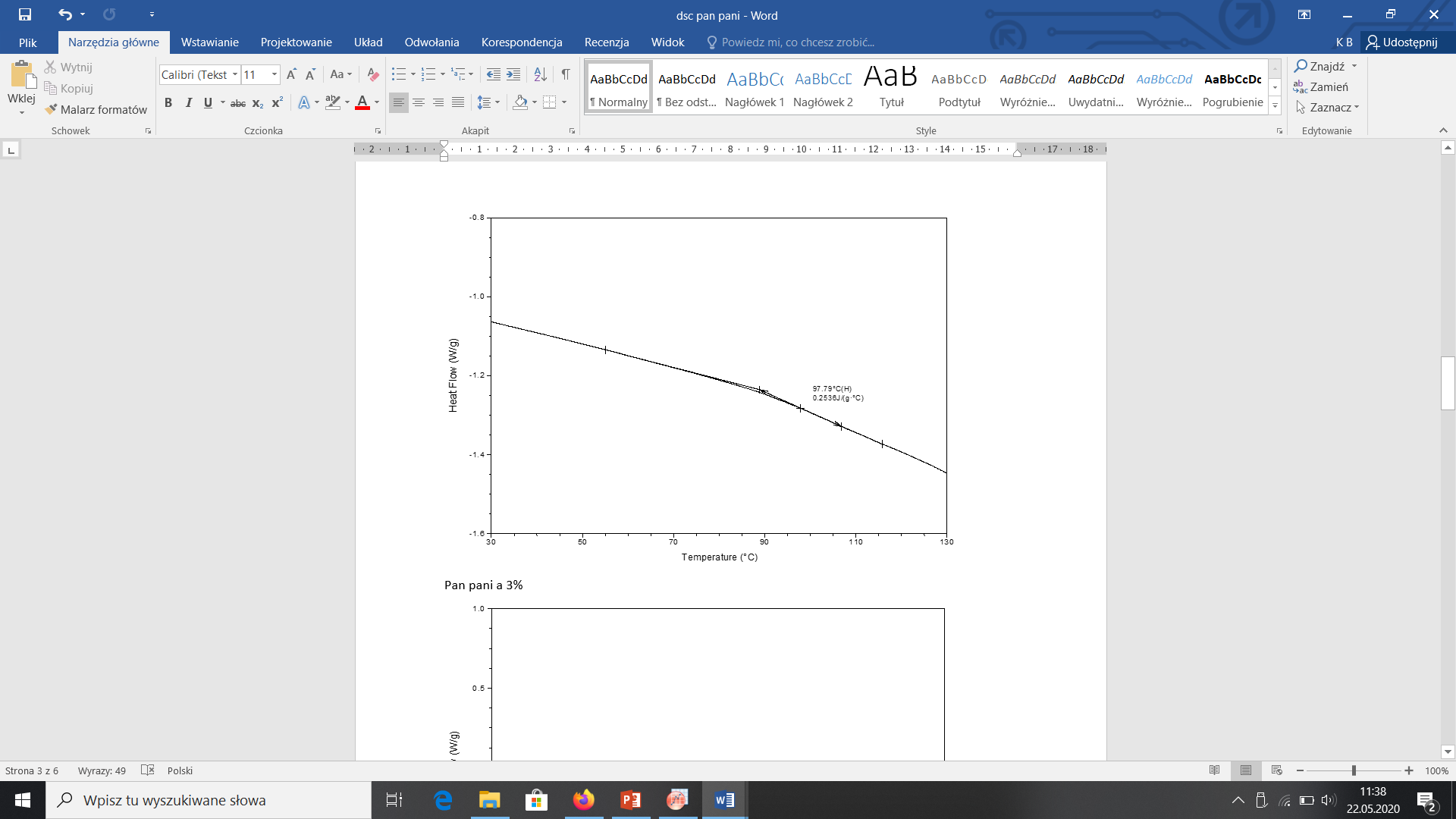


S 10 DSC graph of PAN-PANI (C)3%

| Temperature (°C) | Storage modulus (Pa) | | | | | |
| --- | --- | --- | --- | --- | --- | --- |
|  | PAN–PANI A(1%) | PAN–PANI A(3%) | PAN–PANI B(1%) | PAN–PANI B(3%) | PAN–PANI C(1%) | PAN–PANI C(3%) |
| -150 | 1,17823E7 | 1,16679E7 | 1,16718E7 | 1,16764E7 | 1,15404E7 | 1,15328E7 |
| -120 | 1,125E7 | 1,1084E7 | 1,10898E7 | 1,11E7 | 1,10228E7 | 1,10794E7 |
| -90 | 1,22382E7 | 1,19461E7 | 1,2221E7 | 1,23164E7 | 1,21002E7 | 1,20501E7 |
| -60 | 1,25588E7 | 1,24271E7 | 1,23914E7 | 1,25465E7 | 1,23061E7 | 1,23232E7 |
| -30 | 1,33767E7 | 1,32351E7 | 1,3213E7 | 1,33682E7 | 1,31416E7 | 1,31081E7 |
| 0 | 1,34715E7 | 1,33195E7 | 1,32802E7 | 1,33603E7 | 1,31937E7 | 1,3192E7 |
| 30 | 1,36978E7 | 1,35606E7 | 1,3713E7 | 1,34502E7 | 1,35569E7 | 1,35356E7 |
| 60 | 1,44058E7 | 1,43604E7 | 1,43488E7 | 1,422E7 | 1,4419E7 | 1,4365E7 |
| 90 | 1,63379E7 | 1,62394E7 | 1,62502E7 | 1,61241E7 | 1,62166E7 | 1,61506E7 |
| 120 | 1,77252E7 | 1,79147E7 | 1,79512E7 | 1,75705E7 | 1,7607E7 | 1,7449E7 |
| 150 | 1,91734E7 | 1,91709E7 | 1,92471E7 | 1,9094E7 | 1,8999E7 | 1,88442E7 |
| 180 | 2,03084E7 | 2,02944E7 | 2,03293E7 | 1,99535E7 | 1,98876E7 | 1,95549E7 |
| 210 | 2,06432E7 | 2,05629E7 | 2,05555E7 | 2,03023E7 | 2,0057E7 | 1,96903E7 |

S11 Storage modulus versus temperature of conductive composites.
